# The Effect of RAGE-Diaph1 Signaling Inhibition on the Progression of Peripheral Neuropathy in Diabetic Mice

**DOI:** 10.3390/ijms262211182

**Published:** 2025-11-19

**Authors:** Kamila Zglejc-Waszak, Agnieszka Korytko, Bernard Kordas, Andrzej Pomianowski, Bogdan Lewczuk, Joanna Wojtkiewicz, Krzysztof Wąsowicz, Izabella Babińska, Konark Mukherjee, Judyta Karolina Juranek

**Affiliations:** 1 Department of Anatomy and Histology, School of Medicine, Collegium Medicum, University of Warmia and Mazury in Olsztyn, 10-085 Olsztyn, Poland; 2 Department of Human Physiology and Pathophysiology, School of Medicine, Collegium Medicum, University of Warmia and Mazury in Olsztyn, 10-085 Olsztyn, Polandjoanna.wojtkiewicz@uwm.edu.pl (J.W.); 3 Internal Medicine Department, Faculty of Veterinary Medicine, University of Warmia and Mazury in Olsztyn, 10-719 Olsztyn, Poland; 4 Department of Histology and Embryology, Faculty of Veterinary Medicine, University of Warmia and Mazury in Olsztyn, 10-719 Olsztyn, Poland; lewczukb@uwm.edu.pl; 5 Department of Pathophysiology, Forensic Veterinary Medicine and Administration, Faculty of Veterinary Medicine, University of Warmia and Mazury in Olsztyn, 10-719 Olsztyn, Poland; 6 Department of Genetics, University of Alabama at Birmingham, Birmingham, AL 35233, USA

**Keywords:** cytoskeleton, type 1 diabetes, neuropathy, sciatic nerve, Diaph1, RAGE

## Abstract

Diabetic peripheral neuropathy (DPN) is a serious consequence of prolonged hyperglycemia and contributes to the morbidity associated with diabetes. Hyperglycemia enhances the non-enzymic glycation of proteins and the accumulation of Advanced Glycation End Products (AGEs). We employed a diabetic mouse model lacking both Diaph1 and RAGE to elucidate the role of RAGE-Diaph1 signaling in the pathogenesis of DPN. We demonstrate that simultaneous deletion of Diaph1 and RAGE did not change the course or the intensity of hyperglycemia-induced weight loss in mice. However, abrogating RAGE-Diaph1 signaling affects actin cytoskeleton remodeling rates in nerve axons by altering the ratio of the actin-regulating molecules cofilin and profilin. Our experimental results suggest that the loss of RAGE-Diaph1 signaling protects neurons from hyperglycemic conditions. We observed a beneficial effect of abolishing RAGE-Diaph1 signaling on the axonal structure of neuropathic nerves. In addition, we observed that abolishing RAGE-Diaph1 signaling improved motor nerve conduction velocity in the sciatic nerves of hyperglycemic mice. Our data indicate that RAGE-Diaph1 signaling is likely enhanced in chronic hyperglycemia, resulting in aberrant actin dynamics in nerve axons. These defective actin dynamics play a key role in the progression of DPN, leading to structural and functional loss in peripheral nerves.

## 1. Introduction

Peripheral neuropathy (DPN) is one of the most common neurometabolic complications of diabetes, affecting up to 90% of all diabetic patients worldwide [1]. A range of high glucose-mediated effects may contribute to the pathogenesis of diabetic neuropathy, including cytoskeleton protein glycation, oxidative stress, enhanced perineuronal inflammation and axonal transport alteration [1,2].

Multiple theories by which glycation exacerbates diabetic neuropathy exist; the most intriguing facet is the concept that not only do nerve fibers degenerate in diabetic neuropathy, but also, their attempts at regeneration are impaired [3,4,5,6,7,8]. It has been found that regenerative sprouts, although produced, fail to survive [1], and it is plausible that abnormally glycated collagen in the endoneurium of nerve trunks may act as a physical barrier to impede elongation of regenerating axonal sprouts. In addition, the glycation of scaffolding, cytoskeleton components such as actin, profilin or cofilin may impair axonal ability to regrow and flourish by affecting axonal transport, causing neuronal dysfunction and impeding proper axonal migration and attachment [2,3,4,9,10]. It is crucial to tease out the cell-intrinsic cytoskeletal mechanism from the microenvironmental collagen-glycation-mediated disruption of nerve regeneration to validate the appropriate therapeutic target for DPN. Unlike the endoneurium-glycation-mediated impairment of neurite outgrowth, the cell-intrinsic changes are likely to be mediated by RAGE (Receptor for Advanced Glycation End Products) and its intracellular interactor, Diaph1 (Diaphanous Related Formin 1)-mediated signaling.

New insights into the RAGE/Diaph1 signaling pathway revealed that it may regulate actin dynamics via a complex interplay of Diaph1 with actin regulatory proteins profilin 1 (PFN1) and cofilin (CFL) [2,3,4,10]. Experimental evidence suggests that in chronic hyperglycemia, formation of RAGE/Diaph1 molecular complex in peripheral nerves leads to an aberrant actin cytoskeletal dynamic which contributes to DPN progression [11,12]. Our previous studies indicated that proteins involved in actin cytoskeleton dynamics, Diaph1, PFN1 and ACTB, were down-regulated in the sciatic nerve of wild-type mice during long-term diabetes [2]. Furthermore, we observed perturbations in nerve conduction velocity as well as morphometry of sciatic nerve harvested from diabetic mice [2]. The function of actin within nerve fibers until now has been mostly studied in the maintenance of the axon initiation segment, formation of growth cones and synaptic dynamics [10,11,12]. However, a recent discovery of axonal actin rings has renewed interest in the role of actin in axonal morphology and transport. Defects in actin dynamics in peripheral nerve fibers in DPN are therefore likely to contribute to its progression.

Previous results have implicated the Diaph1/RAGE signaling pathway in diabetic neuropathy [2]. Furthermore, rodent studies have shown that blocking RAGE-Diaph1 interactions delays neuropathic malfunctions [1,3]. Until now, no study, however, has sought to directly examine the effect of complete abrogation of RAGE/Diaph1 expression on nerve axon cytoskeleton. Herein, for the first time, we have studied how simultaneous double deletion of RAGE and Diaph1 genes affects the axonal cytoskeletal proteins in the course of DPN in mice. We discovered that simultaneous deletion of Diaph1 and AGER (RAGE encoding gene) prevents the reduction in beta-actin (ACTB) expression in nerve fibers and preserves its co-localization with CFL. Diaph1 and AGER double deletion also improved sciatic nerve morphology in diabetic mouse models. Thus, our study provides clear evidence that the cell-intrinsic cytoskeletal aberration downstream of RAGE/Diaph1 signaling plays a crucial role in histopathological changes associated with DPN.

## 2. Results

**Figure 1:**
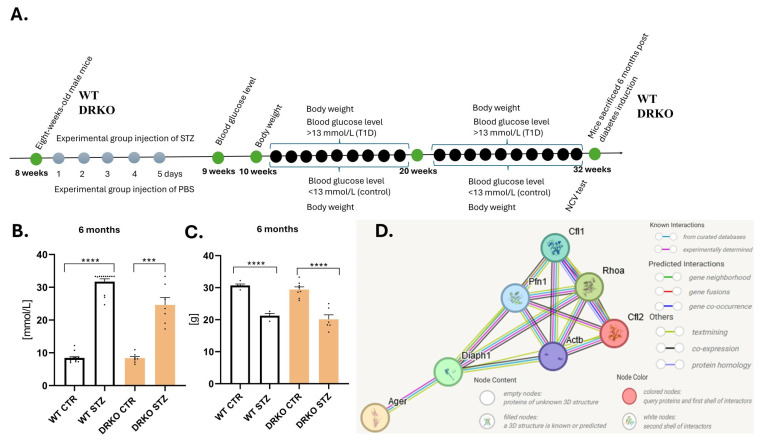
Experimental design to investigate diabetic neuropathy. (**A**). Schematic of the experimental design. The treatment plan, the age/sex of the animals and the specific assays are indicated within the figure. The green circles indicate specific milestones in the experiment, the purple circles indicate the prediabetic time points and the black circle indicates diabetic timepoints. (**B**). Blood glucose level of mice of indicated genotype (WT = wild type, DRKO = Diaph1/RAGE knockout) either treated with PBS (control = CTR) or streptozotocin (STZ) is plotted as mean ± SEM. (**C**). Weight of mice (in grams) of indicated genotype and treatment (control = CTR) or streptozotocin (STZ) is plotted as mean ± SEM. Each black dot in the bar graphs represents a mouse. Statistical analyses are performed as described in the Materials and Methods section. *** *p* ≤ 0.001, **** *p* ≤ 0.0001. The number of individuals (n) is represented by dots (signs on the graph). (**D**). Bioinformatic analysis of studied proteins. Proteins form a common network, confirming their interdependence when studying diabetic neuropathy in mice. String database has been used to depict evidence of physical interaction between RAGE/Diaph1 with molecules involved in actin dynamics including ACTB (actin), PFN1 (profilin 1), CFL (cofilin) and RhoA.

Four groups of mice (WT control, WT T1D, DRKO control and DRKO T1D, Figure 1A) were compared in terms of coping with the disease. Mice from all groups were constantly monitored [2].

### 2.1. Effect of Diaph1 and AGER Deletion on Body Mass and Blood Glucose Levels in Mice

To examine the effect of simultaneous Diaph1 and AGER/RAGE deletion on progression of DPN we first needed to verify whether a lack of these molecules could alter the course of murine T1D itself (Figure 1B,C). Blood glucose levels were already more than twice as high after six months in the diabetic groups (WT and DRKO) compared to the control (*p* ≤ 0.0001 and *p* ≤ 0.001, respectively, Figure 1B). Mice with simultaneous deletion of the Diaph1 and AGER/RAGE developed hyperglycemic levels as quickly and to the same level as their WT counterparts (Figure 1B). Weight loss was observed in diabetic mice in both cases (*p* ≤ 0.0001, Figure 1C).

### 2.2. Potential Role of Diaph1, RAGE, ACTB, PFN1, CFL1 and CFL2 as Well as RhoA Proteins in Progression of DPN

Novel insights into RAGE/Diaph1 signaling reveal that it is mediated by actin dynamics in the nervous system [4]. UniProt database revealed that PFN1, ACTB, CFL1 and RhoA are involved in actin cytoskeleton of the peripheral nerve (https://www.uniprot.org/ (accessed on 20 August 2025). It is important to note that previous studies linked PFN1, ACTB and CFL1 as well as RhoA proteins with RAGE/Diaph1 signaling pathways [1,2,4]. In silico analysis indicated that all of them are connected in one network and thus may contribute to the development of DPN (Figure 1D, Table S2).

### 2.3. Double Deletion of Diaph1 and AGER Impacts Molecules Involved in Actin Dynamics in T1D Mouse Model

Studies have shown that blocking Diaph1 interaction with RAGE reduces the progression of neuropathy [1,3]. Deleting AGER/RAGE contributes to delaying neurodegenerative diseases in mice [7,8,9]. Our aim was to investigate how double deletion of RAGE and Diaph1 contributes to the improvement of nerve conduction, sciatic nerve morphometry and the abundance of proteins involved in the actin cytoskeleton.

### 2.4. Immunoreactivity of ACTB, PFN1, CFL and RhoA upon Deletion of Diaph1 and AGER in T1D Mice

**Figure 2:**
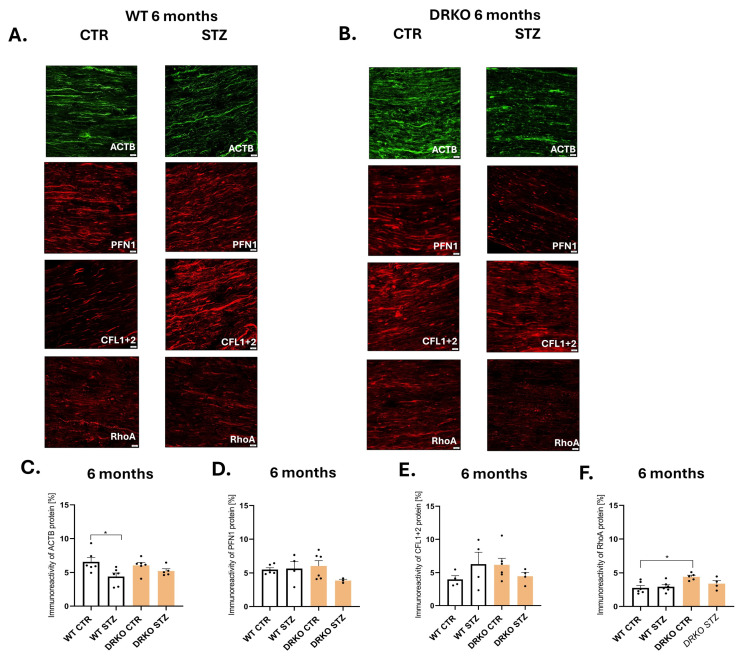
Effect of hyperglycemia and RAGE-Diaph1 signaling on actin dynamic pathway. (**A**,**B**). Representative images from immunofluorescence experiments with selected antibody in WT mice and treatment (CTR = PBS treated, STZ = streptozotocin treated). (**C**). Representative immunofluorescence images with selected antibody and treatment in DRKO mice (CTR = PBS treated, STZ = streptozotocin treated). Scale bar = 20 µm. (**D**–**F**). Quantitation of immunoreactivity intensity of selected antigen in WT and DRKO mice. The total percentage of staining area was calculated per ROI for four technical replicates and four sections of tissues for each of the biological replicates (number of samples) within each group of animals. Experiments were performed 6 months after STZ treatment. Controls were treated with PBS. Data is plotted as mean ± SEM. Each black dot in the bar graph represents a mouse. Statistical analyses are performed as described in the Materials and Methods section. * *p* ≤ 0.05., The number of individuals (n) is represented by dots (signs on the graph).

On account of the evidence linking Diaph1 and AGER to actin cytoskeleton malfunctions [2,3,4,5], and thus DPN, we examined the levels of potential protein candidates (Figure 1D) regulated by Diaph1 and AGER/RAGE in the sciatic nerves harvested from mice with six months of T1D (Figure 2A–F). Immunofluorescence staining revealed that at all examined time points, ACTB, PFN1, CFL and RhoA proteins were present in the mouse sciatic nerve (Figure 2A,B). Results revealed that during long-term T1D, the immunoreactivity of ACTB was decreased in the sciatic nerve of WT (Diaph1^+/+^AGER^+/+^) mice compared to their respective controls (*p* ≤ 0.05, Figure 2C). Further, we observed elevated amounts of RhoA in the peripheral nerves harvested from DRKO control mice as well as the reduced amount of RhoA in tissue of WT control mice (*p* ≤ 0.05, Figure 2F).

### 2.5. Deletion of Diaph1 and AGER in Mice Alters ACTB and PFN1 Ratio in Sciatic Nerve

**Figure 3:**
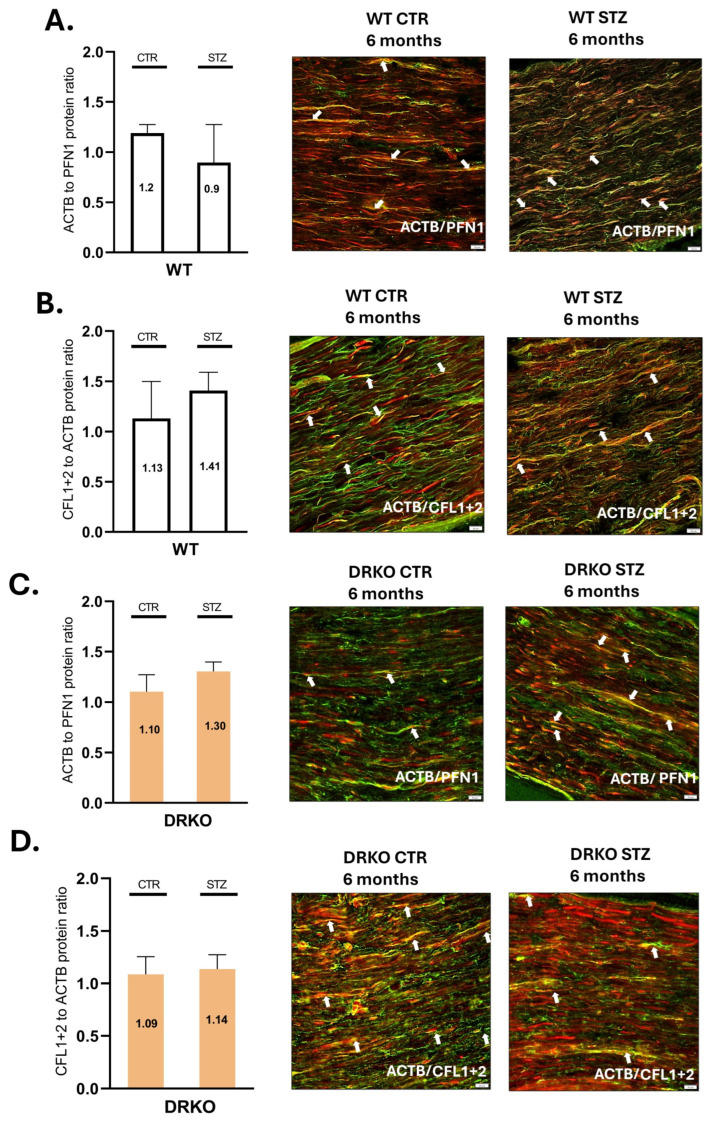
Effect of hyperglycemia and RAGE-Diaph1 signaling on the ratio of actin to regulators of actin polymerization. (**A**). Left panel: Ratio of actin to profilin has been plotted as a bar graph in non-diabetic and diabetic wild-type mice. Right panel: Representative images of actin/profilin co-labeled nerves of nondiabetic (control) and diabetic (STZ) WT mice at 6 months. (**B**). Left panel: Ratio of actin to cofilin has been plotted as a bar graph in non-diabetic and diabetic WT mice. Right panel: Representative images of actin/cofilin co-labeled nerves of nondiabetic (control) and diabetic (STZ) WT mice at 6 months. (**C**). Left panel: Ratio of actin to profilin has been plotted as a bar graph in non-diabetic and diabetic DRKO mice. Right panel: Representative images of actin/profilin co-labeled nerves of nondiabetic (control) and diabetic (STZ) DRKO mice at 6 months. (**D**). Left panel: Ratio of actin to cofilin has been plotted as a bar graph in non-diabetic and diabetic DRKO mice. Right panel: Representative images of actin/cofilin co-labeled nerves of nondiabetic (control) and diabetic (STZ) DRKO mice at 6 months. Scale bar = 20 µm. Four biological replicates, each containing four technical replicates, were performed. White arrows indicate colocalization (yellow color.). Green indicates ACTB, red indicates CFL1+2 or PFN1.

Evidence indicated that PFN1 plays an essential role in actin elongation [2,3,4,5,13,14,15,16,17]. Moreover, profilin binds to actin at a ratio of one to one and thus regulates actin nucleation [17], and the elevated concentrations of PFN1 may cause malfunctions in actin polymerization [17]. Hence, we specifically tested whether simultaneous deletion of Diaph1 and AGER in mice led to malfunctions in ACTB and PFN1 ratio in the T1D sciatic nerve (Figure 3A,C). We observed double staining for ACTB and PFN1 in sciatic nerve fibers (Figure 3A,C). Our results showed that the ratio of ACTB and PFN1 was decreased in T1D sciatic nerve of WT mice but not in DRKO mice (Figure 3A,C, Table S3).

### 2.6. Deletion of Diaph1 and AGER in Mice Alters CFL and ACTB Ratio in Sciatic Nerve

Studies revealed that the cofilin and actin ratio may play a crucial role in neuronal development and degeneration [10] and that the elevated cofilin/actin (CFL/ACTB) ratio may trigger neuronal depolymerization [10]. We demonstrated immunoreactivity of ACTB and CFL in the mouse sciatic nerve (Figure 3B,D). We observed that long-term T1D triggers an increased CFL/ACTB ratio in sciatic nerves harvested from diabetic WT mice vs. respective control groups (Figure 3B). However, in mice with simultaneous deletion of Diaph1 and AGER gene, we noted that the ratio of cofilin to actin remained at the physiological level (approximately 1:1, Figure 3D). Together, these observations suggest a close association between Diaph1 and AGER gene simultaneous deletion and cofilin/actin ratio in T1D sciatic nerve (Figure 3B,D, Table S3).

### 2.7. Effect of Deletion of Diaph1 and AGER on Primary Mouse Neuronal Cells

**Figure 4:**
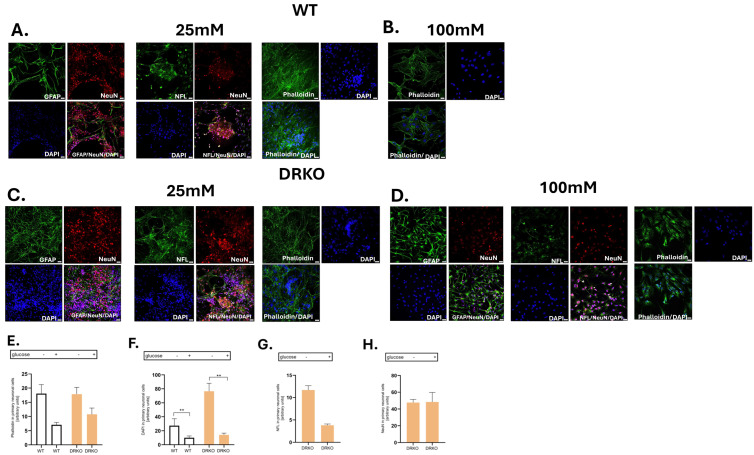
Effect of hyperglycemia and RAGE-Diaph1 signaling on in vitro neuronal survival. (**A**). Neural cultures from WT mice in media containing 25 mM glucose labeled for indicated molecules. GFAP stands for glial fibrillary acidic protein, DAPI or 4′,6-diamidino-2-phenylindole is a DNA binding dye and labels the nucleus, NeuN or neuronal nuclei is specific to neuronal nucleus, NFL is neurofilament and phalloidin labels actin filaments. (**B**). Neural cultures from WT mice in media containing 100 mM glucose labeled for indicated molecules. (**C**). Neural cultures from DRKO mice in media containing 25 mM glucose labeled for indicated molecules. (**D**). Neural cultures from DRKO mice in media containing 100 mM glucose labeled for indicated molecules. Scale bar = 20 µm. Four biological replicates, each containing four technical replicates, were performed. (**E**–**H**). Fluorescence in arbitrary units has been quantitated for indicated molecules and plotted as a bar graph. Glucose level (−) 25 mM and (+) 100 mM is indicated. The genotypes of neural culture WT and DRKO are provided. ** *p* ≤ 0.01.

Finally, we wanted to verify that the changes we observed in nerve fibers were neuronal in origin. We confirmed the presence of primary neuronal cells with positive Neurofilament light (NFL) and neuronal nuclei (NeuN) staining (Figure 4A–D and Figure S2). Nevertheless, phalloidin staining indicated that F-actin filaments (phalloidin) are better maintained in DRKO compared to the WT mice (no differences in *p*-value; Figure 4D,E). Furthermore, the number of DAPI-positive cells was reduced at high-glucose levels (*p* ≤ 0.01; Figure 4A–D,F). Our data indicates that very high levels of glucose leads to a decrease in the number of neurons in DRKO mice, as evidenced by decreased staining for NFL and NeuN (no differences in *p*-value; Figure 4C,D,G,H). Overall, our data presented here indicates that RAGE/Diaph1 signaling is likely to play a role in altering actin dynamics in nerve fibers in response to hyperglycemia (Table S4). Therefore, we next investigated the morphology of axons in T1D mouse model that lacked Diaph1 and AGER.

### 2.8. Concomitant Deletion of Diaph1 and AGER Prevents Hyperglycemia-Induced Morphological Changes in Sciatic Nerve

**Figure 5:**
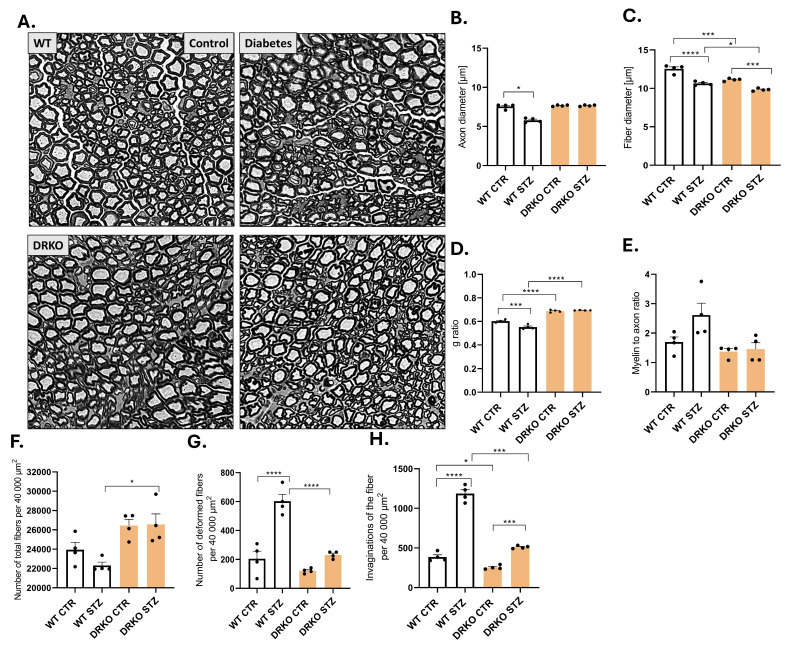
Effect of hyperglycemia and RAGE-Diaph1 signaling on nerve morphology. (**A**). Representative bright-field images of semithin transverse sections of sciatic nerves. Genotype and treatment of mice (control vs. STZ) are shown. The experiments have been performed 6 months after STZ injection. Scale bar = 20 µm. (**B**–**H**). Morphometric quantitation of selected parameters has been performed and plotted as mean ± SEM for control and STZ, WT and DRKO mice, respectively. Statistical analyses are performed as described in the Materials and Methods section. * *p* ≤ 0.05, *** *p* ≤ 0.001, **** *p* ≤ 0.0001. Each black dot in the bar graph represents a mouse.

We specifically analyzed sciatic nerve myelinated fibers in WT and DRKO mice (Figure 5A–F). We examined the axon and fiber diameter and g-ratio as well as myelin to axon ratio, number of total fibers, number of deformed fibers and invaginations per sciatic nerve ROI (Figure 5B–F). These parameters are widely utilized as a functional and structural indicator of optimal axonal morphometry. Alterations from standard values in these parameters may indicate the development of DPN [2,18]. We found that hyperglycemia reduces both the axon and the g ratio in WT mice, but deleting Diaph1 and AGER together prevents this reduction to a similar extent (Figure 5B,C). Again, subsequent Diaph1 and AGER gene deletion reduced fiber diameter in T1D mice compared to controls (Figure 5C, *p* ≤ 0.001). Moreover, T1D induced decreased fiber diameter in WT mice (Figure 5C, *p* ≤ 0.0001). We did not find differences in the g-ratio between non- and diabetic DRKO (Diaph1^−/−^AGER^−/−^) mice (Figure 5D, *p* ≤ 0.05); however, we noted a reduced g-ratio between non- and diabetic WT mice (Figure 5D, *p* ≤ 0.001). We observed that the g-ratio is associated both with T1D and Diaph1 and AGER gene deletion in mice (Figure 5D). We observed that during T1D the number of total fibers was elevated in DRKO mice compared to WT animals (Figure 5F, *p* ≤ 0.05). Our studies showed that the highest number of deformed fibers as well as invaginations were observed in diabetic WT mice (Figure 5F,G). Despite long-term T1D, the number of deformed fibers did not differ in DRKO mice (*p* ≥ 0.05, Figure 5G). Put together, our data indicates that DRKO mice were almost completely devoid of negative signs of hyperglycemia (Figure 5A–F), hence we examined the effect of Diaph1 and AGER deletion on the functionality of the sciatic nerve fibers.

### 2.9. Double Deletion of Diaph1 and AGER Prevents NCV Decline in T1D

**Figure 6:**
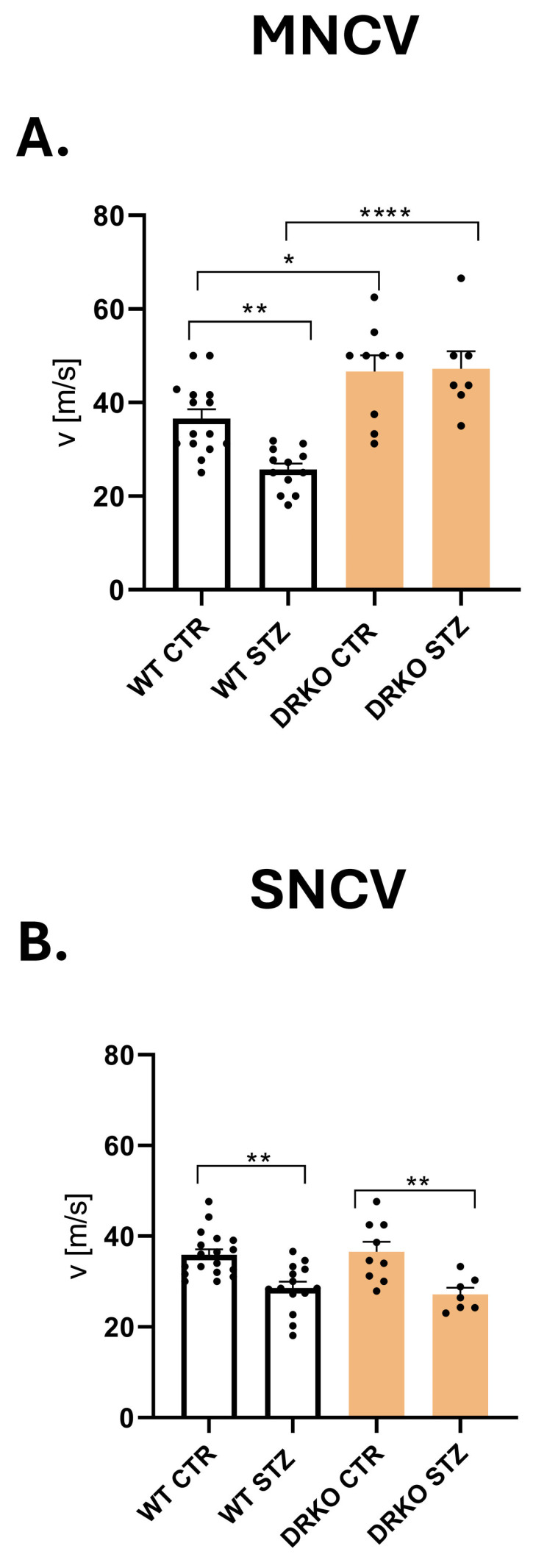
Effect of hyperglycemia and RAGE-Diaph1 signaling on nerve conduction velocity. (**A**). Motor nerve conduction velocity (MNCV) is plotted as mean ± SEM for control and STZ, WT and DRKO mice, respectively. All abbreviations are consistent with previous figures. (**B**). Sensory nerve conduction velocity (SNCV) is plotted as mean ± SEM for control and STZ, WT and DRKO mice, respectively. Statistical analyses are performed as described in the Materials and Methods section. * *p* ≤ 0.05, ** *p* ≤ 0.01, **** *p* ≤ 0.0001. Each black dot in the bar graph represents a mouse.

NCV is the gold standard for diagnosis of DPN. There are strong indications that this is the most reliable test to confirm the presence of neuropathy in a mouse model of the disease [2,19]. Since mice remain under anesthesia, the results of the experiment are not influenced by environmental factors such as noise, smell, or human presence [2,18]. We performed MNCV and SNCV in T1D mice three and six months after STZ-injection (Figure 6A,B). MNCV was significantly decreased after six months of T1D in WT mice when compared with their respective controls (*p* ≥ 0.01, Figure 6A). In contrast to these findings, there were no significant differences in MNCV in diabetic compared to non-diabetic mice with Diaph1 and AGER simultaneous deletion (*p* ≥ 0.05, Figure 6A). Moreover, we observed the elevated levels of MNCV in T1D mice with global deletion of Diaph1 and AGER genes vs. WT mice at six months of diabetes (*p* ≤ 0.0001, Figure 6A). As diabetes progressed, a decrease in SNCV was observed in WT as well as DRKO compared to control groups (*p* ≤ 0.01 in both cases, Figure 6B). Collectively, simultaneous deletion of Diaph1 and AGER genes abolishes MNCV impairment in mice with six months of T1D, potentially eliminating signs of long-term T1D in mice.

## 3. Discussion

Diabetic peripheral neuropathy (DPN) is a common consequence of uncontrolled diabetes and contributes to profound morbidity, including conditions such as diabetic foot [1,2,3,4,5,18,19,20,21,22,23]. DPN is characterized by axonal atrophy and demyelination [1,2,3,4,13,14,15,16,17,24]. Prolonged hyperglycemia likely damages nerves through multiple mechanisms, including microvascular damage, metabolic and oxidative stress, neuroinflammation and signaling by AGE [1,2,3,4,18]. We previously demonstrated that experimental DPN is associated with perturbation of the RAGE/Diaph1 signaling pathway [2,3,4,5]. In this study, we therefore sought to decipher the role of RAGE/Diaph1 signaling in the molecular pathogenesis of DPN.

To avoid pitfalls of parallel and redundant pathways, we utilized DRKO double-knockout mice. The DRKO mice are otherwise phenotypic and readily develop a hyperglycemic condition upon treatment with STZ. Similar to WT mice, they also rapidly lose weight upon induction of hyperglycemia, indicating that not all adverse metabolic effects of hyperglycemia can be negated by abolishing RAGE/Diaph1 signaling. Biochemical and cell-biological studies suggest that Diaph1 may function by regulating the dynamics of actin polymerization (reviewed in detail in Juranek et al. [1] and Zglejc-Waszak et al. [3]). Based on our previous work and the literature [1,2,3,4,5,6,7,8,25,26,27,28], we hypothesized that aberrant RAGE/Diaph1 signaling in hyperglycemia likely alters molecules involved in actin polymerization, thus impacting axonal structure. Actin rings are thought to provide mechanical support for axons [29,30,31,32]. Indeed, we found that actin immunoreactivity is reduced in sciatic nerves under hyperglycemic conditions; furthermore, this reduction is completely abolished in DRKO mice. Our results, therefore, directly implicate RAGE/Diaph1 signaling in the actin dynamics of nerve axons.

Based on our findings, we suggest that in hyperglycemic conditions, RAGE-Diaph1 signaling leads to actin depolymerization via altering its ratio to regulating molecules like cofilin and profilin, resulting in reduced staining intensity in nerves. Incidentally, we also observed an increase in RhoA in DRKO mice. RhoA is known to disinhibit Diaph1 [11,32,33,34], and this observed increase in the absence of Diaph1 may, therefore, be compensatory in nature. However, the increase in RhoA may also have other molecular mechanisms.

Are alternative interpretations of our results possible? Since the mice used in this project lack RAGE and Diaph1 ubiquitously, non-cell-intrinsic mechanisms contributing to improvements in nerve structure and function cannot be ruled out [1]. For example, RAGE-Diaph1 signaling may play a role in diabetes-induced atherosclerosis [35,36,37,38] and could participate in microvascular damage-mediated nerve injuries [39,40]. Similarly, RAGE-Diaph1-mediated signaling also influences endothelial permeability and cell migration [39,40], processes critical for inflammation. An increase in inflammation could exacerbate nerve damage [41,42,43].

However, our biochemical findings provide direct evidence for the alteration of actin dynamics in nerve fibers. We suggest that this is a crucial mechanism through which RAGE-Diaph1 signaling contributes to the development of DPN.

Additional lines of evidence support this notion. Deletion of RAGE/Diaph1 does not uniformly improve the manifestations of DPN. While MNCV improves in DRKO mice, the reduction in SNCV is not rescued. We contend that non-cell-intrinsic mechanisms, such as microvascular damage or inflammation, are likely to affect both MNCV and SNCV, rather than selectively influencing MNCV. Furthermore, the improvement in MNCV is observed even under normoglycemic conditions, where the likelihood of vascular pathology or neuroinflammation is minimal [1,2,3].

Similarly, the improvement in nerve morphology in DRKO mice is not uniform. The number of nerve fibers (myelinated axons) and the morphology of nerve fibers both display improvements in DRKO mice [44]. However, much of this improvement is also observed under normoglycemic conditions. It is conceivable that even under physiological conditions, steady-state signaling via the RAGE-Diaph1 axis is detrimental to nerves, potentially contributing to age-dependent neuropathies [1,38,40,45,46,47,48,49,50]. This detrimental role may become further exacerbated under chronic hyperglycemia. Alternatively, it is feasible that RAGE-Diaph1 signaling has a beneficial role in peripheral nerve pruning, eliminating excess axonal branches. In fact, even under normoglycemic conditions, we observed a reduction in nerve fiber diameter and an increase in the g-ratio of nerve fibers, indicating greater preservation of thinner fibers [1,2,40]. It is possible that both these mechanisms coexist. Further experiments are required to address these processes.

Finally, in DRKO mice, the hyperglycemia-dependent reduction in axon (unmyelinated) diameter is rescued. Overall, it appears that the physiological molecular functions of RAGE-Diaph1 signaling may overlap with its role in diabetic conditions [35,36,37,38,45,51,52,53]. Experiments utilizing neuron-specific inducible knockout models of RAGE-Diaph1 are required to further elucidate both the physiological and pathological roles of this crucial signaling pathway [54,55,56,57,58]. The physiological role of RAGE-Diaph1 signaling also warrants investigation in the brain, as a homozygous mutation in Diaph1 has been associated with microcephaly [1,2,3,56,57,59,60], highlighting its critical role in early brain development.

Overall, both pharmacological and genetic studies in animal models suggest that inhibiting RAGE/Diaph1 signaling may alleviate certain aspects of DPN, raising the specter of future translational studies. Studies indicating that imaging modalities such as diffusion tensor imaging may serve as an excellent indicator of histopathological damage in DPN are likely to further enhance the feasibility of such studies [61].

## 4. Materials and Methods

### 4.1. Animals

All experiments were approved by the Local Ethics Committee of Experiments on Animals in Olsztyn, Poland; decision no. 57/2019. Mouse lines were created by the CRISPR-Cas9 method at the International Institute of Molecular and Cell Biology in Warsaw, Poland. To verify the knock-out of Diaph1, genotyping was performed using specific Diaph1 primers (Forward: GCATTGCTGTCTCTTACACA, Reverse: TCAACTTAGGAGACCACACA). Three Diaph1 products at 504 bp and 313 bp as well as 201 bp confirmed Diaph1 knock-out (DKO). Subsequently, to verify the knock-out of AGER/RAGE, genotyping was performed using specific primers (Forward: TTGCTCTATGGGGTGAGACA, Reverse: GTAGACTCGGACTCGGTAG). Two AGER/RAGE products at 380 bp and 278 bp confirmed AGER knock-out (RKO) [8]. To generate the animals for this study, DKO (Diaph1^−/−^ AGER^+/+^) males were mated with RKO (Diaph1^+/+^AGER^−/−^) females to obtain breeders with the following genotype Diaph1^−/+^ AGER^+/−^. Then, Diaph1^−/+^AGER^+/−^ males were mated with Diaph1^−/+^AGER^+/−^ females to obtain the genotypes analyzed in the study (DRKO; Diaph1^−/−^AGER^−/−^).

Eight-week-old male mice (C57BL/6 (wild type—WT), DRKO (C57BL/6 background)) were randomly divided into control and experimental groups per defined time points (Figure 1A). T1D was induced by intraperitoneal injection of streptozotocin (STZ, 50 mg/kg; Sigma-Aldrich, St. Louis, MO, USA) for five consecutive days (Figure 1A). Mice were anesthetized with a mixture of ketamine (300 mg/kg) and xylazine (30 mg/kg) 24 weeks post the last STZ injection (6 months of diabetes, Figure 1A). Animals were euthanized with limited suffering. The number of animals has been marked in the figures.

### 4.2. Nerve Conduction Velocity and Morphometric Studies

In order to confirm DPN, we performed motor and sensory nerve conduction velocity (MNCV, SNCV) tests as well as morphometric analysis of the sciatic nerve [2,18,19]. Briefly, morphometric analyses [2] were performed within the region of interest (ROI, sciatic nerve cross-section area of 40,000 μm^2^).

### 4.3. Bioinformatic Analysis of Studied Proteins

We aimed to show that the studied proteins participate in the dynamics of the actin cytoskeleton and together they form a common network of interactions. The list of proteins was uploaded to STRING v. 12 to identify biological processes, molecular functions, cellular components, KEGG pathways and node of the STRING network [20].

### 4.4. Immunofluorescence Staining

The presence of ACTB, PFN1, CFL (we used an antibody detecting CFL1 in non-muscle cells and CFL2 in skeletal and cardiac muscles; CFL1 + 2) and RhoA proteins (Table 1) as well as co-localization and co-expression between ACTB and PFN1 or ACTB and CFL in sciatic nerve sections harvested from WT, DRKO mice, was investigated using a two-day procedure for immunofluorescence staining [2,8]. Slides were mounted with a mounting medium with DAPI (Sigma-Aldrich, USA) and examined under a fluorescent microscope (Olympus IX83, Tokyo, Japan). Immunofluorescence was quantified using ImageJ v. 1.54k as described previously [2,8]. Briefly, we standardized all images, automatically converted the positive signal into the corresponding range of gray values and the percentage of immunoreactivity was counted in four technical replicates for each biological replicate. We confirmed the specificity of the antibodies using a negative control (Figure S1).

### 4.5. Primary Mouse Neuronal Cell Culture

Primary mouse neurons were obtained from WT and DRKO embryos (E17–18) [21,22,23]. Cells were plated onto µ-Slide 8-Well high Poly-L-Lysine coated chamber slides (Ibidi, Gräfelfing, Germany) density of 5.21 × 10^2^ neurons per well (growth area 1 cm^2^) [21]. Primary murine neurons were cultured in B-27 Neuron Plating Medium and maintained at 37 °C with 5% CO_2_ [22,23]. Two days after plating, Cytosine β-D-arabinofuranoside (10 μM, Sigma-Aldrich, USA) was added to the culture and maintained for 12 h to prevent astrocyte proliferation. After 12 h, Plating Medium was removed, cells were washed once with Hanks’ Balanced Salt solution and Neuron Feeding Medium (Table S1) was added [22,23]. Control neurons remained in the medium containing a physiological concentration of glucose (25 mM) for neurons for the next five days prior to further manipulations [13,14,15,16,22,23]. Cells for glucose treatment were cultured in a medium containing 100 mM glucose [13,14,15,16]. During the experiment, the high-glucose medium (100 mM) was replaced with a fresh medium every day [13,14,15,16].

### 4.6. Immunostaining of Cultured Neurons

Neuronal cells were washed in phosphate-buffered saline (PBS, EURX, Poland) and then fixed in fixation buffer. The immunofluorescent staining procedure (Table 1) was performed according to the protocol described in Cymerys et al. [23].

### 4.7. Statistical Analyses

Statistical analyses and graphs were performed using GraphPad Prism 9.1.0. (San Diego, CA, USA). We performed the Shapiro–Wilk test. The Shapiro–Wilk test is used to check the normality of the data distribution. Based on the normality of the data distribution, we chose parametric one-way as well as two-way ANOVA with Tukey’s post hoc or non-parametric test and the Kruskal–Wallis test for statistical testing. Fold change was calculated for selected datasets [2]. We met the minimum sample size requirements for statistical analysis [44]. We assumed that alpha = 0.05 and power = 80%.

## 5. Conclusions

In summary, this is the first study to examine the effect of RAGE/Diaph1 double deletion on the pathogenesis of DPN. Here, we established that deletion of these molecules abolishes the alteration in actin dynamics observed in nerves under hyperglycemic conditions. Furthermore, we demonstrated an overall positive effect of these deletions on neuronal health in vitro as well as on nerve structure in vivo under hyperglycemic conditions. Thus, the RAGE-Diaph1 pathway remains a viable therapeutic target for DPN. However, further studies are required to carefully distinguish its physiological and pathological functions.

## Figures and Tables

**Table 1:** Antibodies used for immunofluorescence staining.

Primary Antibodies				
Antigen	Code	Species	Working Dilution	Supplier
Immunofluorescence staining				
Sciatic nerve				
**ACTB**	251006	Chicken	1:200	Synaptic System, Göttingen, Germany
**PFN1**	Ab232020	Rabbit	1:100	Abcam, Cambridge, UK
**CFL1 + 2**	Ab131519		1:100	
**RhoA**	Ab187027		1:100	
**Primary mouse neuronal cell culture**				
**NeuN**	Ab177487	Rabbit	1:100	Abcam, Cambridge, UK
**GFAP**	173006	Chicken	1:700	Synaptic System, Göttinge, Germany
**NFL**	171006		1:200	
**Immunofluorescence staining**				
**Reagents**	Code	Working Dilution		Supplier
**Goat anti-Rabbit IgG (H + L) Highly Cross-Adsorbed Secondary Antibody, Alexa Fluor™ Plus 594**	A32740	1: 2000		ThermoFisher, Waltham, MA, USA
**Goat anti-Chicken IgY, Alexa Fluor 488**	A-11039			
**Fluoroshield™ with DAPI**	F6057-20 ML	3–4 drops of mounting medium directly on top of the specimen		Sigma-Aldrich, St. Louis, MO, USA

## Data Availability

The original contributions presented in this study are included in the article/Supplementary Material. Further inquiries can be directed to the corresponding author.

## Supplementary Materials

The supporting information can be downloaded at https://www.mdpi.com/article/10.3390/ijms262211182/s1.

## Abbreviations

The following abbreviations are used in this manuscript:

ACTB	beta actin
AGE	advanced glycation end products
CFL	cofilin
DPN	diabetic peripheral neuropathy
Diaph1	Diaphanous Related Formin 1
DKO	Diaph1 knockout mouse
DRKO	double knockout (Diaph1^−/−^AGER^−/−^)
FH1	Formin Homology 1
MNCV	motor nerve conduction velocity
NCV	Nerve conduction velocity
NFL	Neurofilament light
NeuN	neuronal nuclei
PKC	Protein kinase C
PBS	phosphate-buffered saline
RAGE	Receptor for Advanced Glycation End Products
RKO	AGER knock-out
ROI	region of interest
SNCV	Sensory nerve conduction velocity
STZ	streptozotocin
T1D	Type 1 diabetes
WT	Wild type
