# Supplementary material for: The Effect of RAGE-Diaph1 Signaling Inhibition on the Progression of Peripheral Neuropathy in Diabetic Mice
Source: Int J Mol Sci. 2025 Nov 19;26(22):11182. doi: 10.3390/ijms262211182 (PMC12653783; doi:10.3390/ijms262211182)
Supplement: Supplementary file 1 [file ijms-26-11182-s001.zip › S_Table_1(1).pdf]

Supplementary Table S1. Components of the culture medium

**B-27 Neuron Plating Medium**		
**Reagent**	**Catalog no.**	**Supplier**
Neurobasal™ Medium	21103049	Thermo Fisher, USA
B-27™ Supplement (50X)	17504044	
Glutamic acid (10 mmol/L; 1.8 mg/mL)	G2128	Sigma Aldrich, USA
L-glutamine (200 mmol/L)	25030149	Thermo Fisher, USA
Fetal bovine serum	A3160401	
Horse serum	26050070	
Penicillin-Streptomycin (10,000 U/mL)	15140122	
Cytosine β-D-arabinofuranoside	C1768	Sigma-Aldrich, USA
**Neuron Feeding Medium**		
**Reagent**	**Catalog no.**	**Supplier**
Neurobasal™ Medium	21103049	Thermo Fisher, USA
B-27™ Supplement (50X)	17504044	
L-glutamine (200 mmol/L)	25030149	
Fetal bovine serum	A3160401	
Horse serum	26050070	
Penicillin-Streptomycin (10,000 U/mL)	15140122	
**Neuron Feeding Medium with glucose**		
**Reagent**	**Catalog no.**	**Supplier**
Neurobasal™ Medium	21103049	Thermo Fisher, USA
B-27™ Supplement (50X)	17504044	
L-glutamine (200 mmol/L)	25030149	
Fetal bovine serum	A3160401	
Horse serum	26050070	
Penicillin-Streptomycin (10,000 U/mL)	15140122	
D-(+)-Glucose solution	G8769	Sigma Aldrich, USA
