# Supplementary material for: The Effect of RAGE-Diaph1 Signaling Inhibition on the Progression of Peripheral Neuropathy in Diabetic Mice
Source: Int J Mol Sci. 2025 Nov 19;26(22):11182. doi: 10.3390/ijms262211182 (PMC12653783; doi:10.3390/ijms262211182)
Supplement: Supplementary file 1 [file ijms-26-11182-s001.zip › S_Table_2(1).pdf]

Suplementarny Table S2. Functional analysis revealed that the studied proteins could be categorized within biological process, molecular function and cellular component terms as well as biological pathway hierarchy

Biological Process (Gene Ontology)				
GO-term	description	count in network	strength	false discovery rate
GO:0030043	Actin filament fragmentation	2 of 4	3.19	0.0013
GO:0098885	Modification of postsynaptic actin cytoskeleton	2 of 10	2.8	0.0041
GO:0030836	Positive regulation of actin filament depolymerization	2 of 13	2.68	0.0052
GO:0045792	Negative regulation of cell size	2 of 14	2.65	0.0054
GO:0051014	Actin filament severing	2 of 15	2.62	0.0057
GO:0008154	Actin polymerization or depolymerization	3 of 61	2.19	0.0010
GO:0032232	Negative regulation of actin filament bundle assembly	2 of 40	2.19	0.0193
GO:0010596	Negative regulation of endothelial cell migration	2 of 57	2.04	0.0289
GO:0001738	Morphogenesis of a polarized epithelium	2 of 57	2.04	0.0289
GO:0051496	Positive regulation of stress fiber assembly	2 of 58	2.03	0.0291
GO:0030838	Positive regulation of actin filament polymerization	2 of 63	2.0	0.0314
GO:1901224	Positive regulation of NIK/NF-kappaB signaling	2 of 71	1.94	0.0366
GO:0032231	Regulation of actin filament bundle assembly	3 of 116	1.91	0.0041
GO:0000281	Mitotic cytokinesis	2 of 78	1.9	0.0405
GO:0071677	Positive regulation of mononuclear cell migration	2 of 84	1.87	0.0441
GO:0008064	Regulation of actin polymerization or depolymerization	4 of 178	1.85	0.00085
GO:0043525	Positive regulation of neuron apoptotic process	2 of 89	1.85	0.0478
GO:1902905	Positive regulation of supramolecular fiber organization	4 of 192	1.81	0.00085
GO:0030833	Regulation of actin filament polymerization	3 of 158	1.77	0.0058
GO:0051495	Positive regulation of cytoskeleton organization	4 of 217	1.76	0.00085
GO:0008360	Regulation of cell shape	3 of 165	1.75	0.0061
GO:0007015	Actin filament organization	4 of 276	1.66	0.0010
GO:1902107	Positive regulation of leukocyte differentiation	3 of 211	1.65	0.0102
GO:0007163	Establishment or maintenance of cell polarity	3 of 226	1.62	0.0115
GO:0050870	Positive regulation of T cell activation	3 of 246	1.58	0.0145
GO:0010632	Regulation of epithelial cell migration	3 of 248	1.58	0.0146
GO:0030036	Actin cytoskeleton organization	6 of 555	1.53	3.03e-05
GO:0022898	Regulation of transmembrane transporter activity	3 of 301	1.49	0.0222
GO:0007162	Negative regulation of cell adhesion	3 of 325	1.46	0.0256
GO:2000146	Negative regulation of cell motility	3 of 332	1.45	0.0256
GO:0051493	Regulation of cytoskeleton organization	5 of 572	1.44	0.00085
GO:0050808	Synapse organization	3 of 338	1.44	0.0256
GO:2000147	Positive regulation of cell motility	5 of 622	1.4	0.00085
GO:0060562	Epithelial tube morphogenesis	3 of 375	1.4	0.0311
GO:0071900	Regulation of protein serine/threonine kinase activity	3 of 378	1.39	0.0312
GO:0045862	Positive regulation of proteolysis	3 of 384	1.39	0.0314
GO:0045785	Positive regulation of cell adhesion	4 of 518	1.38	0.0054
GO:0034330	Cell junction organization	4 of 547	1.36	0.0058
GO:0002009	Morphogenesis of an epithelium	4 of 542	1.36	0.0058
GO:1904062	Regulation of cation transmembrane transport	3 of 420	1.35	0.0374
GO:0030335	Positive regulation of cell migration	4 of 595	1.32	0.0069
GO:0071417	Cellular response to organonitrogen compound	4 of 610	1.31	0.0073
GO:0010959	Regulation of metal ion transport	3 of 462	1.31	0.0441
GO:0034762	Regulation of transmembrane transport	4 of 641	1.29	0.0085
GO:0043269	Regulation of ion transport	4 of 784	1.2	0.0153
GO:0033043	Regulation of organelle organization	6 of 1231	1.18	0.00085
GO:0071495	Cellular response to endogenous stimulus	5 of 1133	1.14	0.0043
GO:0051130	Positive regulation of cellular component organization	5 of 1213	1.11	0.0054
GO:1901701	Cellular response to oxygen-containing compound	4 of 1154	1.03	0.0374
GO:0048699	Generation of neurons	4 of 1259	1.0	0.0462
GO:0030030	Cell projection organization	4 of 1259	1.0	0.0462
GO:0048468	Cell development	6 of 2044	0.96	0.0036
GO:0051128	Regulation of cellular component organization	7 of 2546	0.93	0.00085
GO:0007399	Nervous system development	6 of 2190	0.93	0.0042
GO:0009888	Tissue development	5 of 1902	0.91	0.0222
GO:0051049	Regulation of transport	5 of 1998	0.89	0.0256
GO:0008104	Protein localization	5 of 2005	0.89	0.0256
GO:0071310	Cellular response to organic substance	5 of 2191	0.85	0.0314
GO:0009653	Anatomical structure morphogenesis	5 of 2406	0.81	0.0407
GO:0016043	Cellular component organization	7 of 5482	0.6	0.0172
GO:0048856	Anatomical structure development	7 of 5513	0.6	0.0176
GO:0048522	Positive regulation of cellular process	7 of 5948	0.56	0.0256

	Molecular Function (Gene Ontology)			
GO-term	description	count in network	strength	false discovery rate
GO:0003779	Actin binding	4 of 459	1.43	0.0162
GO:0008092	Cytoskeletal protein binding	6 of 1033	1.26	0.00038

	Cellular Component (Gene Ontology)			
GO-term	description	count in network	strength	false discovery rate
GO:0032587	Ruffle membrane	3 of 96	1.99	0.0032
GO:0016363	Nuclear matrix	3 of 127	1.87	0.0036
GO:0032432	Actin filament bundle	2 of 95	1.82	0.0394
GO:0030027	Lamellipodium	3 of 215	1.64	0.0043
GO:0005938	Cell cortex	4 of 351	1.55	0.0032
GO:0031252	Cell leading edge	4 of 471	1.42	0.0036
GO:0015629	Actin cytoskeleton	4 of 510	1.39	0.0036
GO:0098978	Glutamatergic synapse	4 of 522	1.38	0.0036
GO:0005911	Cell-cell junction	3 of 553	1.23	0.0495
GO:0098794	Postsynapse	4 of 801	1.19	0.0071
GO:0030424	Axon	4 of 847	1.17	0.0083
GO:0043005	Neuron projection	6 of 1721	1.04	0.0032
GO:0098590	Plasma membrane region	4 of 1433	0.94	0.0495
GO:0005856	Cytoskeleton	6 of 2401	0.89	0.0036
GO:0030054	Cell junction	5 of 2232	0.84	0.0202
GO:0043232	Intracellular non-membrane-bounded organelle	7 of 4826	0.66	0.0042

	KEGG Pathways			
pathway	description	count in network	strength	false discovery rate
mmu05133	Pertussis	3 of 73	2.11	0.00023
mmu04520	Adherens junction	2 of 68	1.96	0.0100
mmu04810	Regulation of actin cytoskeleton	6 of 211	1.95	2.07e-09
mmu05100	Bacterial invasion of epithelial cells	2 of 71	1.94	0.0100
mmu04666	Fc gamma R-mediated phagocytosis	2 of 85	1.87	0.0120
mmu04933	AGE-RAGE signaling pathway in diabetic complications	2 of 99	1.8	0.0145
mmu04670	Leukocyte transendothelial migration	2 of 113	1.74	0.0171
mmu04360	Axon guidance	3 of 175	1.73	0.0020
mmu04611	Platelet activation	2 of 120	1.72	0.0176
mmu05135	Yersinia infection	2 of 122	1.71	0.0176
mmu04510	Focal adhesion	3 of 196	1.68	0.0021
mmu04015	Rap1 signaling pathway	3 of 208	1.65	0.0021
mmu05132	Salmonella infection	3 of 212	1.64	0.0021
mmu05418	Fluid shear stress and atherosclerosis	2 of 142	1.64	0.0210
mmu04921	Oxytocin signaling pathway	2 of 145	1.63	0.0210
mmu04530	Tight junction	2 of 152	1.61	0.0210
mmu05205	Proteoglycans in cancer	2 of 196	1.5	0.0325
mmu05170	Human immunodeficiency virus 1 infection	2 of 224	1.44	0.0399

Explain:

Count In Network:

The first number indicates how many proteins in your network are annotated with a particular term. The second number indicates how many proteins in total (in your network and in the background) have this term assigned. You can click on the numbers to see the network view of the gene sets behind them.

Strength:

Log10(observed / expected). This measure describes how large the enrichment effect is. It’s the ratio between i) the number of proteins in your network that are annotated with a term and ii) the number of proteins that we expect to be annotated with this term in a random network of the same size.

False Discovery Rate:

This measure describes how significant the enrichment is. Shown are p-values corrected for multiple testing within each category using the Benjamini–Hochberg procedure.
