# Supplementary material for: The Effect of RAGE-Diaph1 Signaling Inhibition on the Progression of Peripheral Neuropathy in Diabetic Mice
Source: Int J Mol Sci. 2025 Nov 19;26(22):11182. doi: 10.3390/ijms262211182 (PMC12653783; doi:10.3390/ijms262211182)
Supplement: Supplementary file 1 [file ijms-26-11182-s001.zip › S_Table_3(1).pdf]

Supplementary Table S3. ACTB, PFN1, CFL1+2, RhoA regulation in sciatic nerve harvested from mice with deletion of *Diaph1* and *AGER*. Supplementary data to Fig. 2 and Fig. 3

**Protein/Substance**	**Genotype**	**Fold change (diabetes/control)**	**Regulation diabetes vs. control**
**PFN1**	Diaph1$^{+/+}$ AGER$^{+/+}$	1.03	approximately 1, the same level as in the control
	Diaph1$^{-/-}$ AGER$^{-/-}$	0.64	down
**CFL1+2**	Diaph1$^{+/+}$ AGER$^{+/+}$	1.58	**up**
	Diaph1$^{-/-}$ AGER$^{-/-}$	0.72	down
**ACTB**	Diaph1$^{+/+}$ AGER$^{+/+}$	0.67	down
	Diaph1$^{-/-}$ AGER$^{-/-}$	0.86	down
**RhoA**	Diaph1$^{+/+}$ AGER$^{+/+}$	1.06	**up**
	Diaph1$^{-/-}$ AGER$^{-/-}$	0.77	down

**Genotype**	**Type 1 diabetes**	**Ratio CFL1+2 to ACTB**	**Fold change (diabetes/control)**	**Regulation diabetes vs. control**
Diaph1$^{+/+}$ AGER$^{+/+}$	-	1.13	1.25	**up**
Diaph1$^{+/+}$ AGER$^{+/+}$	+	1.41		
Diaph1$^{-/-}$ AGER$^{-/-}$	-	1.09	1.04	approximately 1, the same level as in the control
Diaph1$^{-/-}$ AGER$^{-/-}$	+	1.14		

**Genotype**	**Type 1 diabetes**	**Ratio ACTB to PFN1**	**Fold change (diabetes/control)**	**Regulation diabetes vs. control**
Diaph1$^{+/+}$ AGER$^{+/+}$	-	1.2	0.75	down
Diaph1$^{+/+}$ AGER$^{+/+}$	+	0.9		
Diaph1$^{-/-}$ AGER$^{-/-}$	-	1.1	1.18	**up**
Diaph1$^{-/-}$ AGER$^{-/-}$	+	1.3		
