# Supplementary material for: The Effect of RAGE-Diaph1 Signaling Inhibition on the Progression of Peripheral Neuropathy in Diabetic Mice
Source: Int J Mol Sci. 2025 Nov 19;26(22):11182. doi: 10.3390/ijms262211182 (PMC12653783; doi:10.3390/ijms262211182)
Supplement: Supplementary file 1 [file ijms-26-11182-s001.zip › S_Table_4(1).pdf]

Supplementary Table S4. Phallidin (F-actin), DAPI, NFL, NeuN regulation in primary neuronal cells harvested from mice with deletion of *Diaph1* and *AGER*. Supplementary data to Fig. 4

| **Protein/Substance** | **Genotype** | **Fold change (high glucose/control)** | **Regulation high glucose vs. control** |
|---|---|---|---|
| **Phalloidin** | Diaph1$^{+/+}$ AGER$^{+/+}$ | 0.39 | down |
| | Diaph1$^{-/-}$ AGER$^{-/-}$ | 0.6 | down |
| **DAPI** | Diaph1$^{+/+}$ AGER$^{+/+}$ | 0.37 | down |
| | Diaph1$^{-/-}$ AGER$^{-/-}$ | 0.18 | down |
| **NFL** | Diaph1$^{-/-}$ AGER$^{-/-}$ | 0.33 | down |
| **NeuN** | Diaph1$^{-/-}$ AGER$^{-/-}$ | 1.01 | approximately 1, the same level as in the control |

High glucose 100 mM, control 25 mM
