# Supplementary figures and images for: The Effect of RAGE-Diaph1 Signaling Inhibition on the Progression of Peripheral Neuropathy in Diabetic Mice
Source: Int J Mol Sci. 2025 Nov 19;26(22):11182. doi: 10.3390/ijms262211182 (PMC12653783; doi:10.3390/ijms262211182)

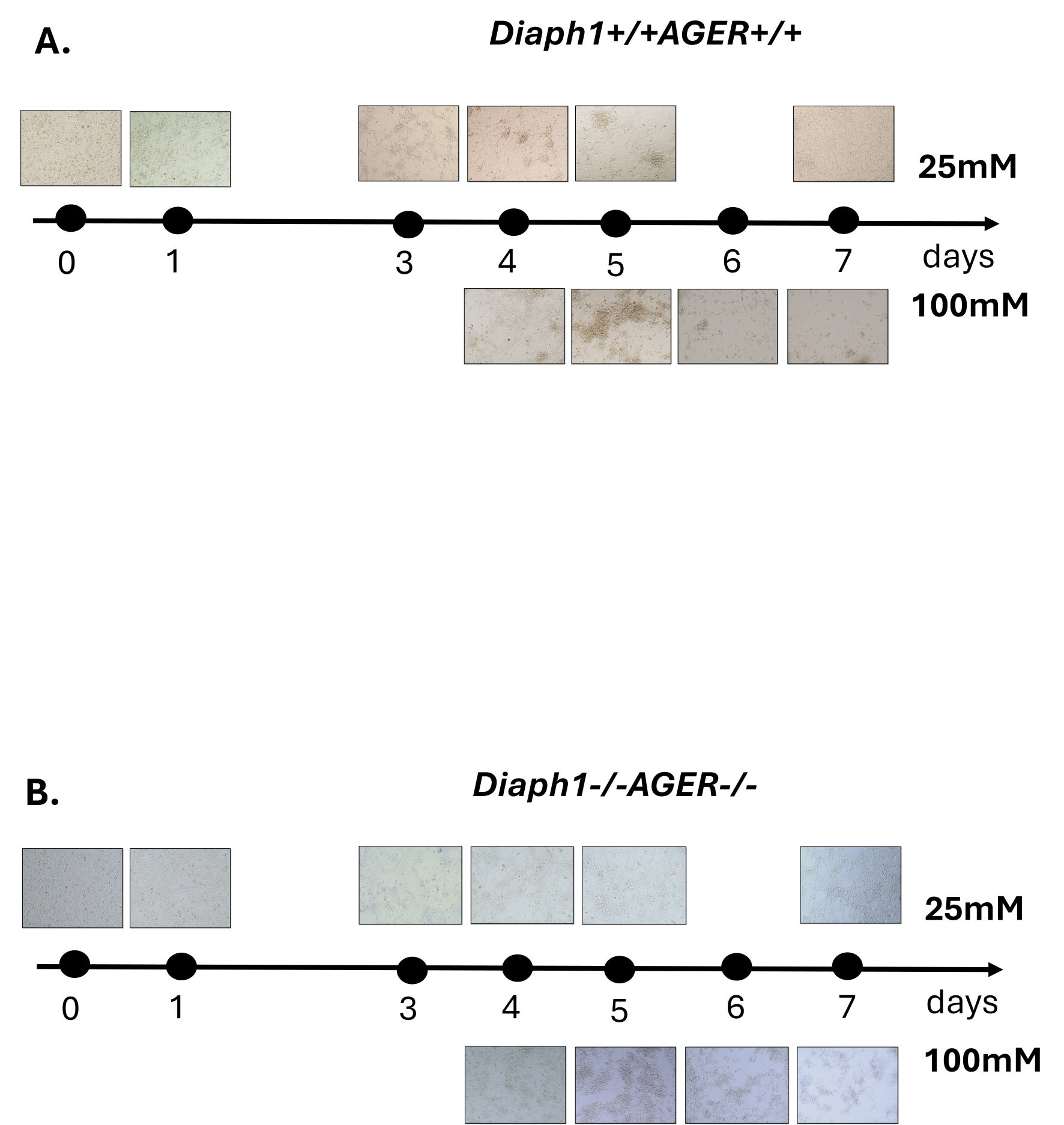

Supplement: Supplementary file 1 [file ijms-26-11182-s001.zip › Supplementary_Fig.2(1).tif]

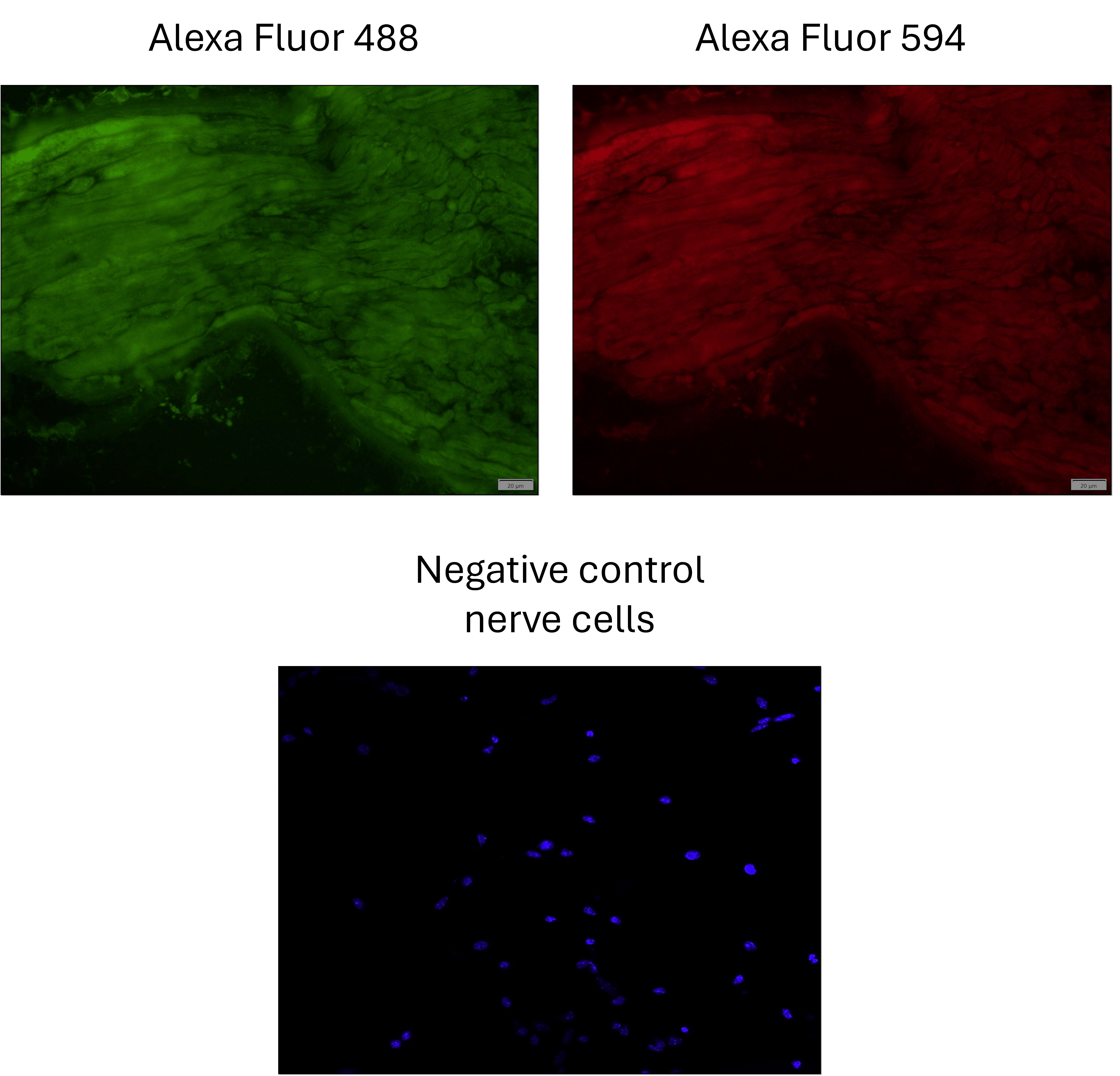

Supplement: Supplementary file 1 [file ijms-26-11182-s001.zip › Supplementary_Fig._1(1).tif]
